# Evaluation of unilateral cage-instrumented fixation for lumbar spine

**DOI:** 10.1186/1749-799X-5-86

**Published:** 2010-11-11

**Authors:** Ti-Sheng Chang, Jia-Hao Chang, Chien-Shiung Wang, Hung-Yi Chen, Ching-Wei Cheng

**Affiliations:** 1 Department of Bio-industrial Mechatronics Engineering, National Chung Hsing University, Taichung, Taiwan; 2 Department of Neurosurgery, Armed Forces Taichung General Hospital, Taichung, Taiwan; 3 Department of Physical Education, National Taiwan Normal University, Taipei, Taiwan

## Abstract

**Background:**

To investigate how unilateral cage-instrumented posterior lumbar interbody fusion (PLIF) affects the three-dimensional flexibility in degenerative disc disease by comparing the biomechanical characteristics of unilateral and bilateral cage-instrumented PLIF.

**Methods:**

Twelve motion segments in sheep lumbar spine specimens were tested for flexion, extension, axial rotation, and lateral bending by nondestructive flexibility test method using a nonconstrained testing apparatus. The specimens were divided into two equal groups. Group 1 received unilateral procedures while group 2 received bilateral procedures. Laminectomy, facectomy, discectomy, cage insertion and transpedicle screw insertion were performed sequentially after testing the intact status. Changes in range of motion (ROM) and neutral zone (NZ) were compared between unilateral and bilateral cage-instrumented PLIF.

**Results:**

Both ROM and NZ, unilateral cage-instrumented PLIF and bilateral cage-instrumented PLIF, transpedicle screw insertion procedure did not revealed a significant difference between flexion-extension, lateral bending and axial rotation direction except the ROM in the axial rotation. The bilateral group's ROM (-1.7 ± 0. 8) of axial rotation was decreased significantly after transpedicle screw insertion procedure in comparison with the unilateral group (-0.2 ± 0.1). In the unilateral cage-instrumented PLIF group, the transpedicle screw insertion procedure did not demonstrate a significant difference between right and left side in the lateral bending and axial rotation direction.

**Conclusions:**

Based on the results of this study, unilateral cage-instrumented PLIF and bilateral cage-instrumented PLIF have similar stability after transpedicle screw fixation in the sheep spine model. The unilateral approach can substantially reduce exposure requirements. It also offers the biomechanics advantage of construction using anterior column support combined with pedicle screws just as the bilateral cage-instrumented group. The unpleasant effect of couple motion resulting from inherent asymmetry was absent in the unilateral group.

## Introduction

Chronic discogenic back pain caused by degenerative disc disease is a common ailment in the general population [1]. The degenerative often results from arthritic changes in the intervertebral discs, facet joints, and ligaments surrounding the vertebral canal [2]. In clinical practice, lateral recess stenosis and foraminal stenosis may induce nerve root compression which can cause unilateral symptoms. Unilateral PLIF may be satisfactory in patients with unilateral symptom. This study evaluated unilateral fusion in patients with unilateral symptoms.

Posterior lumbar interbody fusion (PLIF) has proven successful for relieving motion-induced discogenic pain and was once considered standard treatment for degenerative disc disease [3-5]. A successful PLIF can restore disc height, decompress the dural sac and nerve roots, immobilize the unstable intervertebral disc, and maintain load-bearing to anterior structure [6]. Bilateral PLIF is associated with increased complication rates [7-9]. Elias and coworkers [7] reported a 15% incidence of dural tear and postoperative radiculopathy. It typically caused by excessive epidural bleeding and prolong or excessive retraction. In 1982, Harm et al. [10] popularized the surgical technique of transforaminal lumbar interbody fusion (TLIF). Its advantages are posterior epidural approach with interbody support and bilateral posterior segmental pedicle screw fixation. Biomechanically, preserving longitudinal ligaments is though to preserve stability, to prevent implant dislocation, and to place a compressive force on adequately sized intervetebral implants [11-13]. This technique also avoids retraction of the ligamentum flavum as well as scarring of the spinal canal. Nevertheless, its long learning curve, prolong radiation exposure and high cost make it unsuitable for health insurance coverage in Taiwan.

Performing unilateral PLIF using a single interbody cage has several advantages. Inserting a single interbody cage through a unilateral approach compromises fewer anatomic structures than two cages inserted through a bilateral approach. However, it is unknown that unilateral PLIF can achieve biomechanical stability as the bilateral PLIF. Suke *et al. *[14] found that unilateral pedicle screw fixation was as effective as bilateral pedicle fixation in lumbar spinal fusion independent of one or two levels. Their conclusions cannot extend to the cage-instrumented PLIF due to the different boundary of decompression (especially facetectomy). Tencer *et al. *[15] demonstrated that two PLIF structural devices produced a greater reduction in torsional stiffness than single PLIF device. Chen *et al. *[16] demonstrated that unilateral fixation with two cage insertion is a feasible alternative in spinal surgery. Wang *et al. *[17] showed that an oblique insertion of a single BAK cage in instrumented PLIF might reduce exposure and enable precise implantation. These articles could not provide enough evidence to support that unilateral fusion can supply similar stability as the bilateral fusion. That is why unilateral PLIF did not become routine procedures in the treatment of degenerative lumbar spine disease. Goel [18] demonstrated that the unilateral plate system causes coupled motions due to its inherent asymmetry and was unlikely to provide sufficient rigidity in fresh cadaveric human spines. Rotational deformity of lumbar spine might develop if inherent asymmetry persisted. They considered complete excision of the disc was required. Unilateral cage-instrumented PLIF might overcome inherent asymmetry. From the previous review, this study was conducted with two aims. One was to know the biomechanical stability between unilateral and bilateral cage-instrumented PLIF. The other was to determine the unpleasant effect of couple motion resulting from inherent asymmetry was present or not in the unilateral group.

## Methods

### Specimen Preparation

Twelve motion segments of L4/5 from twelve sheep lumbar spines were studied for this *in-vitro *investigation. At the time of salvage, the animals were 12-18 months old and weighted 60 kg (53 to 65 kg). Following preparation, the specimens were stored frozen at -20°C then thawed at room temperature for 24 hours prior to testing. Care was taken to completely preserve the bony and ligamentous structures of the locomotor segment of each specimen, and only muscular and fatty tissue was removed. The cranial and caudal vertebrae of each functional spinal unit was anchored with stainless-steel screws and embedded with custom-designed metal fixtures using polyester resin. The intervening segments were left unconstrained.

### Instrumentations

**Figure 1:**
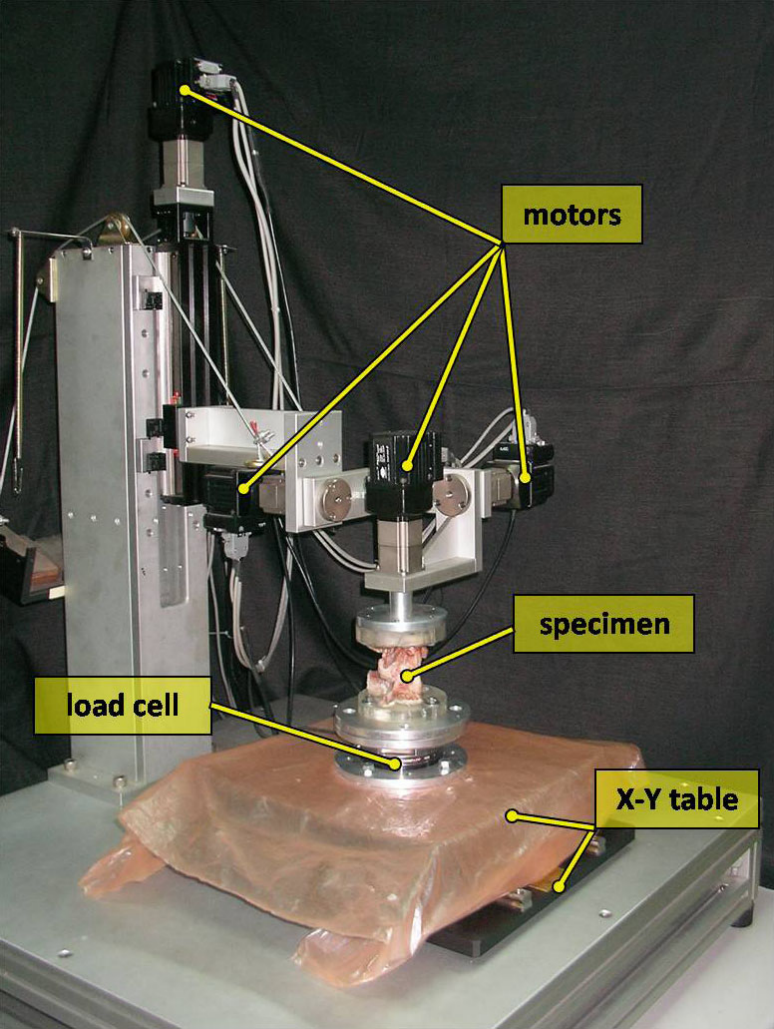
**The testing machine and mounting system with a specimen during performance of a flexibility test**. Various component of this tester is illustrated.

Flexion-extension, left-right lateral bending and left-right axial rotation of each specimen were analyzed by the spinal tester (Figure 1) [19]. The reliability test of this tester was published in the literature [19]. The servo motor (SMART MOTOR 2315D, ANIMATICS, USA) and planetary reduction gearbox (AD042.S2.P1, APEX DYNAMICS, TW) combined to form the drive apparatus. A six-axis load cell (SI-660-60, ATI Industrial automation, USA) measured the moments and the forces during testing procedures. Below the load cell, an X-Y table was used to achieve pure moment on each specimen to prevent shear force. The signal from the load cell was conditioned and connected to a computer to provide a feedback signal for displacement control testing.

### Surgical procedures

The specimens were divided into two equal groups. Group1 included specimens that were tested intact. The following surgical procedures were then performed:

1. left hemilaminectomy,

2. left medial facectomy,

3. left discectomy,

4. left cage insertion (one, 8*8*12 mm), The intervertebral disc and approximately 2-3 mm of endplate and subchronal bone of both vertebral bodies was removed with curret and disc forcep. Care was taken not to place the intervertebral disc under distraction and compression. Custom-made titanium cage (8*8*12 mm, Synthes) was impacted and gently pushed into the intervertebral lumen. The cage was within the intervertebral space about 2 mm below the end plate. No scoliotic deformity was found after this procedure.

5. left transpedicular screw fixation (two screws-4.75*25 cm and one rod- ISOLA system),

The group 2 specimens underwent the same sequence of procedures as the Group 1 except the bilateral sides, which included:

1. total laminectomy,

2. bilateral medial facetectomy,

3. bilateral discectomy,

4. two cage insertion (8*8*12 mm), The intervertebral disc and approximately 2-3 mm of endplate and subchronal bone of both vertebral bodies was removed with curret and disc forcep. Care was taken not to place the intervertebral disc under distraction and compression. Custom-made titanium cage (8*8*12 mm, Synthes) was impacted and gently pushed into the intervertebral lumen. The cage was within the intervertebral space about 2 mm below the end plate. No scoliotic deformity was found after this procedure.

5. bilateral transpedicle screws fixation (four screws-4.75*25 cm and two rods- ISOLA system)

All procedures were performed by a single experienced spinal surgeon and co-author (TSC).

### Testing procedures

The lower vertebrae were centered over the load cell and maintained in neutral position with respect to the set coordinate system described previously [19]. After the specimen was mounted on the spinal tester, left-right lateral bending, flexion-extension and left-right axial rotation of the specimen were conducted at a constant speed of 1°/sec in sequence before and after different surgical procedures. A compressive preload of 100 N was applied. It represented that the spine supports standing posture under external load. The direction was reversed until the moment detected by the load cell reached ±2 Nm. Depending on the specific purpose of the study, the typical load used for biomechanical testing is 6-10 Nm [20-22]. The load applied in the current study was 2 Nm since this load produced the wide range of destruction including facetectomy and discectomy (which are similar to effects observed in clinical practice). In the preliminary investigation, specimen failure occurred at loads exceeding 2 Nm. Krijnen et al. [23] used 1 Nm in a goat model segmental stability with stand-alone cage.

The load cell provided a feedback signal to the computer through RS-232 interface with 40 Hz sampling rate. The load and displacement data were collected and recorded during testing. A real-time graphical display of servo motor angle and applied moment was available during the test.

### Data and Statistical Analysis

The curvilinear regression analysis of force (Y-axis) and displacement (X-axis) with fourth-order polynomials were used to eliminate small variation in force. The ROM of flexion-extension, lateral bending and axial rotation were interpolated at 2 Nm from the moment-angle relation. The NZ is the displacement at the zero-load point measured from the neutral position. The EZ is the displacement from the zero-load point to the maximum load point (2Nm). ROM at the operative L4/5 level was defined as the summation of NZ and EZ at the fifth loading cycle.

To determine the effects of unilateral and bilateral cage-instrumented PLIF differences on outcome measures (ROM, NZ) over the fifth repetition procedures were computed and used for statistical analyses. We used a simple contrast in repeated measurement analysis of variance (rmANOVA) to compare the difference between intact and the rest five procedures. Independent sample T test was performed to compare the difference in ROM and NZ, was calculated by subtracting the previous status from the data after surgical procedure, between unilateral and bilateral cage-instrumented PLIF of flexion-extension, lateral bending and axial rotation. We compared individual procedure through this way. Paired sample *t *test was used to compare the difference in ROM, was calculated by subtracting the intact status from that data after the surgical procedure, between right and left side of lateral bending and axial rotation in the unilateral cage-instrumented PLIF. The statistical data analyses were performed using SPSS for Windows version 13.0 (SPSS. Inc. Chicago, Illinois) software package. A p level less than 0.05 was considered statistically significant.

## Results

**Figure 2:**
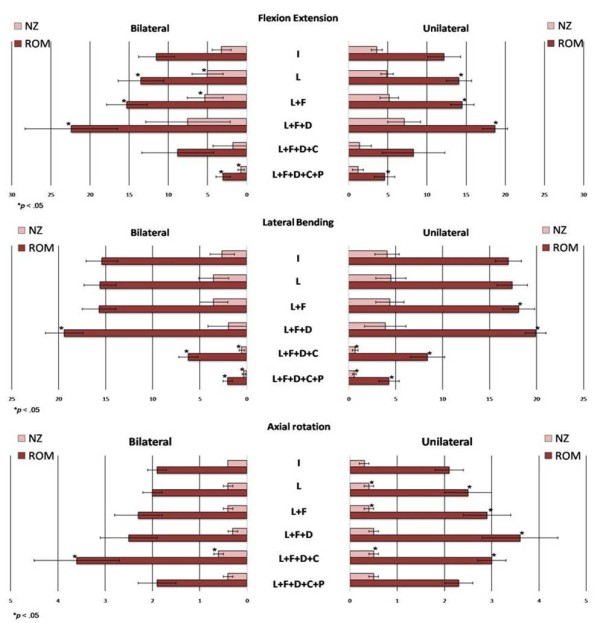
**Effects of destructive and stabilizing procedures on the ROM and NZ of unilateral and bilateral group**. (L: Laminectomy, F: Facetectomy, D: Discetomy, C: Interbody Cage, P: Pedicle screw ).

1. Effects of destructive and stabilizing procedures on the ROM and NZ of unilateral and bilateral group (Figure 2)

Both unilateral and bilateral group, the ROM and NZ of flexion-extension and lateral bending were decreased significantly after transpedicle screw in comparison with intact status. In the axial rotation direction, the ROM and NZ did not decreased after transpedicle screw fixation on both groups.

2. Comparison between unilateral and bilateral group in ROM and NZ (Table 1)

**Table 1:** The difference of ROM and NZ between unilateral and bilateral cage-instrumented PLIF over flexion-extension, lateral bending and axial rotation

Procedures		L		F		D		C		P	
		unilateral	bilateral	unilateral	bilateral	unilateral	bilateral	unilateral	bilateral	unilateral	bilateral
**Flexion Extension (°)**	ROM (°)	1.9 ± 0.9	2.1 ± 0.8	0.5 ± 0.6	1.7 ± 0.7*	4.2 ± 1.1	7.1 ± 7.3	-10.4 ± 4.4	-13.6 ± 3.3	-3.7 ± 3.8	-5.8 ± 4.8
	
	NZ (°)	1.2 ± 0.8	1.8 ± 1.2	0.4 ± 0.6	0.3 ± 0.6	1.9 ± 2.4	2.3 ± 5.3	-5.8 ± 1.6	-5.8 ± 3.9	-0.2 ± 1.5	-1.0 ± 2.8

**Lateral Bending (°)**	ROM (°)	0.4 ± 0.4	0.2 ± 0.5	0.7 ± 0.7	0.1 ± 0.5	1.8 ± 1.6	3.7 ± 1.0*	-11.5 ± 2.6	-13.2 ± 2.2	-4.1 ± 1.7	-4.2 ± 1.7
	
	NZ (°)	0.4 ± 0.8	0.9 ±0.9	0.0 ± 0.8	0.0 ± 0.7	-0.6 ± 2.7	-1.6 ± 1.5	-3.1 ± 2.1	-1.5 ± 1.3	-0.1 ± 0.2	-0.2 ± 0.1

**Rotation (°)**	ROM (°)	0.4 ± 0.3	0.2 ± 0.3	0.4 ± 0.3	0.3 ± 0.4	0.7 ± 0.4	0.2 ± 0.3*	-0.6 ± 0.6	1.1 ± 0.9*	-0.7 ± 0.4	-1.7 ± 0.8*
	
	NZ (°)	0.1 ± 0.0	0.0 ± 0.1*	0.0 ± 0.1	0.0 ± 0.1	0.1 ± 0.1	-0.1 ± 0.1*	0.0 ± 0.2	0.3 ± 0.1*	-0.1 ± 0.1	-0.2 ± 0.1

L: Laminectomy, F: Facetectomy, D: Discetomy, C: Interbody Cage, P: Pedicle screw

Note:

L = (L-I): ROM and NZ of the specimen after laminectomy minus intact status

F = [(I +L+F)-(I+L)]: ROM and NZ of the specimen after facectomy minus laminectomy status

D = [(I+L+F+D)-(I +L+F)]: ROM and NZ of the specimen after discectomy minus facetectomy status

C = [(I+L+F+D+C)-(I+L+F+D)]: ROM and NZ of the specimen after interbody cage insertion minus discectomy status

P = [(I+L+F+D+C+P)-(I+L+F+D+C)]: ROM and NZ of the specimen after tranpedicle screw fixation minus interbody cage insertion status

*: significant: p <.05 compared with unilateral group.

The bilateral group's ROM and NZ of flexion-extension and lateral bending did not significantly differ from transpedicle screw procedure in comparison with the unilateral group. The bilateral group's ROM of axial rotation was decreased significantly after transpedicle screw insertion (-1.7 ± 0.8 vs -0.7 ± 0.4, p < 0.05) procedure in comparison with the unilateral group. The bilateral group's NZ of axial rotation did not significantly differ from transpedicle screw procedure in comparison with the unilateral group.

**Figure 3:**
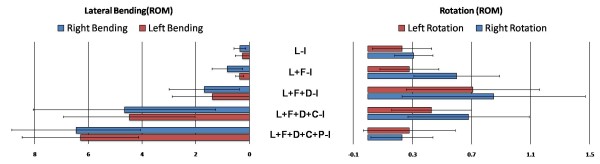
**The difference of ROM between right and left side over lateral bending and axial rotation in the unilateral group**. (L: Laminectomy, F: Facetectomy, D: Discetomy, C: Interbody Cage, P: Pedicle screw ).

3. Symmetry of ROM in the unilateral group: (Table 2, Figure 3)

**Table 2:** The difference of ROM between right and left side over lateral bending and axial rotation in the unilateral group

Procedures	ROM of lateral bending		ROM of axial rotation	
**Lt cage-PLIF**	**Right (°)**	**Left (°)**	**Right (°)**	**Left (°)**
**L-I**	0.35 ± 0.22	0.26 ± 0.26	0.31 ± 0.13	0.23 ± 0.2
**L+F-I**	0.82 ± 0.57	0.36 ± 0.15	0.60 ± 0.29	0.28 ± 0.2
**L+F+D-I**	1.68 ± 1.31	1.38 ± 1.49	0.85 ± 0.62	0.71 ± 0.45
**L+F+D+C-I**	-4.65 ± 3.4	-4.47 ± 2.45	0.68 ± 0.41	0.43 ± 0.27
**L+F+D+C+P-I**	-6.45 ± 2.4	-6.29 ± 2.17	0.23 ± 0.21	0.28 ± 0.31

L: Laminectomy, F: Facetectomy, D: Discetomy, C: Interbody Cage, P: Pedicle screw

Note:

L-I: ROM of the specimen after laminectomy minus intact status

(L+F)-I: ROM of the specimen after facectomy minus intact status

(L+F+D)-I: ROM of the specimen after discectomy minus intact status

(L+F+D+C)-I: ROM of the specimen after interbody cage minus intact status

(L+F+D+C+P)-I: ROM of the specimen after tranpedicle screw minus intact status

The difference in ROM of right lateral bending (6.45 ± 2.40 vs 6.29 ± 2.17) and axial rotation (0.23 ± 0.21 vs 0.28 ± 0.31) did not significantly differ after transpedicle screw as compared with the left side.

## Discussion

The availability of human vertebral column segments is often limited due to the ethical, religious reasons as well as increasing legal restrictions [24]. Therefore, readily available animal vertebral columns were used for testing. Calf, pig and sheep are the three popular species used models to test spinal implants [25,26]. The calf is the species mainly used *in vitro *test for the pedicle screw system. Similar to the calf, the pig is also often used *in vitro *test to measure intradiscal pressure. The sheep and pig is mainly used *in vivo *test for biomechanical experiments [26]. Sheep spines have also been used *in vitro *to study the initial stabilizing effect of spinal implants in the lumbar [27]. The cross-section of six to seven lumbar vertebrae in sheep resembles that in humans [28,29]. The sheep lumbar spine and the human spine also have similar intervertebral disc morphology and spinal musculature anatomy [28]. Sheep pedicles have a smaller diameter, pedicle screw for example need to be shortened to fit the sheep vertebral dimension [25]. Considerable progress in research is apparently needed to achieve satisfactory understanding of the biomechanics of the human lumbar spine before clinical application under the result of animal experiment [25].

*In vitro *testing is an essential for studying spinal mechanics [30,31]. Several parameters may be obtained through biomechanical tests of flexibility to quantify mechanical properties [30]. Such parameters include range of motion (ROM) and neutral zone (NZ). In biomechanical study, the ROM represents the stability of the specimen before and after additional procedures (including destructive and stabilizing procedures). The NZ indicates the laxity around the neutral position of a motion segment as well as residual deformation after removing a defined pure moment load from a motion segment. Mimura *et al. *[31] revealed that, in flexion-extension and lateral bending, ROM decreases and NZ increases during disc degeneration. In the early stage of disc degeneration, ROM increases while in the late stage of disc degeneration, ROM decreases. If only ROM is used as the measurement parameter, misinterpretations are likely. Previous *in vitro *studies indicated that NZ typically increases after experimentally induced injuries [32,33], and that it decreases with the addition of muscle forces and spinal instrumentation [34]. In the ROM and NE, bilateral cage-instrumented group's transpedicle screw insertion procedure did not demonstrate significantly difference in comparison with the unilateral group in flexion-extension, lateral bending and axial rotation direction except the ROM of the rotation. Fortunately, rotation movement occupied little portion of the daily activity. The major portion of daily activity is flexion-extension and lateral bending. Unilateral cage-instrumented PLIF have similar biomechanical stability to bilateral cage-instrumented PLIF in this sheep spine model. Studies have demonstrated increased complications associated with bilateral PLIF [7-9]. Bilateral instrumental fusion technique requires wide dissection, which jeopardizes the function of the paraspinal muscle. The unilateral approach can substantially reduce exposure requirements. This technique is easier than routine bilateral cage-instrumented PLIF. When treating unilateral sciatica patients, the cage can be placed from the symptomatic side so as to avoid retraction of the nerve root and dura sac of the asymptomatic side.

Goel [18] demonstrated the difference in rigidity between unilateral and bilateral instrumentation in fresh cadaveric human spines. They reported that the unilateral plate system causes coupled motions due to its inherent asymmetry and was unlikely to provide sufficient rigidity for decompressive procedures and would therefore require complete excision of the disc. Unilateral instrumentation is unadvised due to the effect of inherent asymmetry. In the current study, no significant differences were shown between right and left side in the lateral bending and axial rotation in the fusion procedures. This method may achieve sufficient stability. The reason is that the inserted cage can be held by the screw-rod system, and the cage can maintain adequate disc space, which is particularly effective for correcting instability associated with degenerative disc disorder.

There are some limitations in this study. First, the paraspinal muscles were remved; however, these muscles had an *in vivo *stabilizing effect therefore, our results probably overestimated the destabilizing effect of the segmental bone and other soft tissue alternation. Second, in this *in vitro *test, the results only reflect acute postoperative stability with relatively few loading cycles and are not necessarily indicative of repetitive loading cycles in the human spine. The biological effects of the potential healing process are unpredictable. Third, subsidence is a complication of cage sinking with loss of normal intervertebral height. The mean subsidence was greater in the one cage model because of the less contact area than the two cage model. An *in vivo *study may be necessary to show whether unilateral group had higher incidence of subsidence or not.

In conclusion, based on the results of this study, unilateral cage-instrumented PLIF and bilateral cage-instrumented PLIF have similar stability in the sheep spine model. The unilateral approach can substantially reduce exposure requirements. It also offers the biomechanics advantage of construction using anterior column support combined with pedicle screws just as the bilateral cage-instrumented PLIF. The unpleasant effect of couple motion resulting from inherent asymmetry was absent in the unilateral group.

## Competing interests

The authors declare that they have no competing interests.

## Authors' contributions

Author contributions to the study and manuscript preparation include the following. Conception and design: TSC, JHC, CWC; Acquisition of data: CSW; Analysis and interpretation of data: TSC, JHC, CWC; Critically revising the article: TSC, JHC; Reviewed final version of the manuscript and approved it for submission: TSC, JHC; Statistical analysis: HYC; Administrative/technical/material support: TSC, JHC, CWC; Study supervision: CWC. All authors read and approved the final manuscript.
